# Optimizing Helmet Use Detection in Construction Sites via Fuzzy Logic-Based State Tracking

**DOI:** 10.3390/s25206487

**Published:** 2025-10-21

**Authors:** Xiaoxiong Zhou, Xuejun Jia, Jian Bai, Xiang Lv, Xiaodong Lv, Guangming Zhang

**Affiliations:** 1 College of Electrical Engineering and Control Science, Nanjing Tech University, Nanjing 211816, China; zhouxx@njtech.edu.cn (X.Z.); jxj1147208@sina.com (X.J.); baijian@njtech.edu.cn (J.B.); lxd@ycit.edu.cn (X.L.); 2 China Construction Second Engineering Bureau Co., Ltd., Beijing 100160, China; lvxiang@mails.jlu.edu.cn; 3 School of Electrical Engineering, Yancheng Institute of Technology, Yancheng 224051, China

**Keywords:** helmet detection, YOLOv5, DeepSORT, fuzzy logic

## Abstract

Automated safety monitoring on construction sites requires precise helmet-status detection and robust multi-object tracking in long, occlusion-rich video sequences. This study proposes a two-stage framework: (i) a YOLOv5 model enhanced with self-adaptive coordinate attention (SACA), which incorporates coordinate-aware contextual information and reweights spatial–channel responses to emphasize head-region cues—SACA modules are integrated into the backbone to improve small-object discrimination while maintaining computational efficiency; and (ii) a DeepSORT tracker equipped with fuzzy-logic gating and temporally consistent update rules that fuse short-term historical information to stabilize trajectories and suppress identity fragmentation. On challenging real-world video footage, the proposed detector achieved a mAP@0.5 of 0.940, surpassing YOLOv8 (0.919) and YOLOv9 (0.924). The tracker attained a MOTA of 90.5% and an IDF1 of 84.2%, with only five identity switches, outperforming YOLOv8 + StrongSORT (85.2%, 80.3%, 12) and YOLOv9 + BoT-SORT (88.1%, 83.0%, 10). Ablation experiments attribute the detection gains primarily to SACA and demonstrate that the temporal consistency rules effectively bridge short-term dropouts, reducing missed detections and identity fragmentation under severe occlusion, varied illumination, and camera motion. The proposed system thus provides accurate, low-switch helmet monitoring suitable for real-time deployment in complex construction environments.

## 1. Introduction

Construction remains one of the most hazardous industries, with falls and head injuries a leading cause of severe and fatal incidents [1,2]. Proactive, site-wide monitoring of workers’ helmet-wearing status is therefore essential. In practice, this requires coupling per-frame helmet-status detection with multi-object tracking (MOT) in long, cluttered videos captured by fixed site cameras, under real-time constraints.

**Figure 1:**
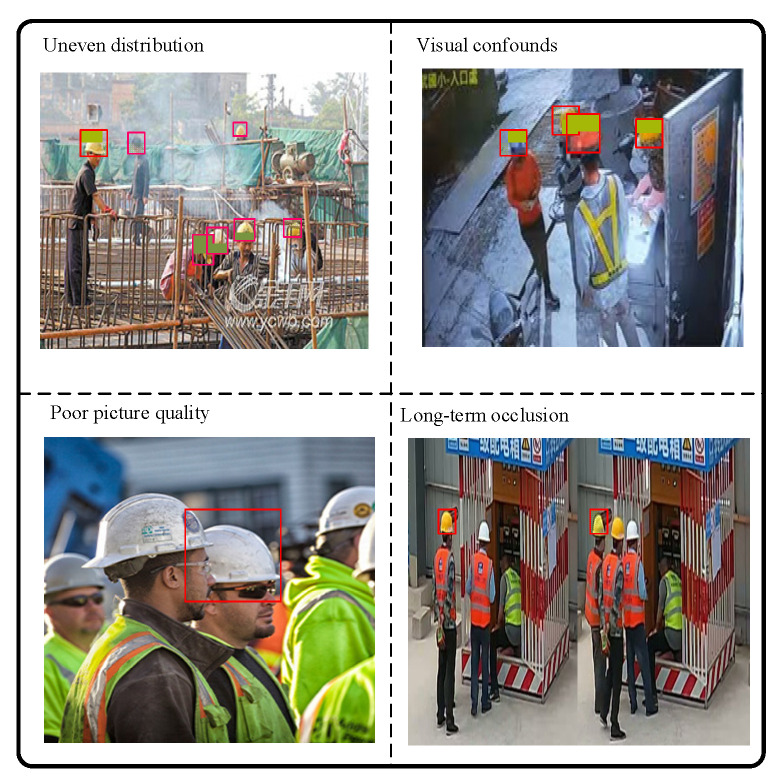
Challenges of helmet wearing detection on construction sites.

Compared with generic pedestrian scenes, construction sites pose distinctive vision challenges (Figure 1): (i) an uneven distribution of workers that are frequently occluded by tools, scaffolds, or body pose, making the head–helmet boundary hard to resolve [3,4]; (ii) visual confounds—caps, hoods, and high-visibility vests with similar colors—produce hard negatives [5]; (iii) motion blur and rapid illumination changes from machinery and outdoor conditions degrade appearance features [6,7]; and (iv) long-duration identity persistence is required as crews enter/exit and re-appear after occlusion, which commonly fragments tracks and triggers identity switches [8,9].

Although many countries have enacted laws requiring construction workers to wear helmets, traditional manual supervision methods have numerous limitations. First, manual inspections often fail to cover the entire construction site and require a large workforce for real-time monitoring, which is both costly and inefficient [10]. Additionally, traditional video surveillance systems typically rely on simple image processing methods that work well in static environments but struggle in complex dynamic environments. In particular, when workers move quickly, change their posture, or experience partial occlusion, these methods often suffer from reduced accuracy, leading to false positives or missed detections [11].

With the rapid development of artificial intelligence and computer vision technologies, video-based safety helmet detection has gradually become an important tool for safety management on construction sites. Most existing helmet detection methods rely on traditional image processing and deep learning technologies such as the YOLO series of object detection models. YOLOv5, as an efficient real-time object detection framework, has an excellent performance in many computer vision tasks. However, in the complex and dynamic environment of construction sites, the traditional YOLOv5 model still faces limitations when dealing with fast-moving workers, occlusion, and complex backgrounds [12]. Furthermore, object tracking algorithms such as DeepSORT are widely used for multi-object tracking tasks but still encounter issues with recognition errors and identity switches when dealing with highly similar targets or occlusions [13].

To address these challenges, this study proposes an improved helmet detection and tracking framework based on YOLOv5 and a fuzzy logic-enhanced DeepSORT algorithm. The main innovations of this work are reflected in the following two key aspects:

(1) Improved YOLOv5 for helmet detection: To enhance detection accuracy in dynamic construction environments, an adaptive spatial and channel attention mechanism was integrated into YOLOv5. This enabled the model to dynamically adjust its attention regions based on contextual information, thereby improving the precise identification of targets. Particularly in cases where the workers’ heads are occluded or moving rapidly, the improved YOLOv5 demonstrated superior capability in detecting helmet presence.

(2) In the tracking phase, DeepSORT was enhanced with a fuzzy logic module that dynamically adjusted the target-matching weights by combining intersection over union (IoU) and appearance features. When targets experience occlusion or visual similarity, fuzzy logic enables a more flexible and adaptive matching strategy, improving the tracking accuracy and stability. Furthermore, to address occlusion and target loss in dynamic environments, a temporal processing-based multi-frame information fusion method was introduced. By integrating information from consecutive frames, this approach mitigates the impact of false or missed detections in individual frames, thereby enhancing the model’s robustness and continuity in complex scenarios.

The remainder of this paper is organized as follows. Section 2 reviews related research progress; Section 3 presents the proposed method and technical framework, with a focus on the application of fuzzy logic to the DeepSORT tracking algorithm; Section 4 describes the dataset and experimental design; Section 5 reports the experimental results; and Section 6 discusses the findings, summarizes the contributions, and outlines future research directions.

## 2. Related Works

### 2.1. Helmet Wearing Detection

Compared with generic pedestrian detection, helmet-wearing detection on active construction sites is harder for three reasons: the effective head region is small and frequently truncated or occluded by tools and scaffolds; caps, hoods, and high-visibility garments introduce look-alike distractors; and illumination shifts and motion blur are common in long, cluttered videos [14,15]. Early pipelines built on color/shape heuristics, background subtraction, or classical descriptors (e.g., HOG/SVM) are lightweight but degrade sharply under pose change, scaffold clutter, and lighting variation, producing unstable precision/recall in real sites [16,17,18]. Modern CNN detectors—both two-stage and one-stage, with YOLO-family models widely used for real-time PPE analytics—improve the per-frame accuracy through multiscale feature pyramids, dense heads, and better optimization [19,20,21,22]; Attention and transformer augmentations further emphasize the small head region and suppress background noise [23,24,25,26]. Progress has also come from anchor-free heads, improved label-assignment strategies, and focal/IoU-style losses that raise the sensitivity to small objects and address class imbalance [27,28,29] as well as feature alignment/refinement modules and hard-negative mining that reduce misalignment and PPE color confounds [30,31,32]. Nevertheless, per-frame decisions remain brittle under partial visibility, crowded scenes, and rapid lighting/pose changes, which continue to trigger false alarms and misses in practice [33,34,35].

Beyond detecting helmets per se, many studies infer helmet-wearing status by encoding the head–helmet spatial relationship—via pose/keypoint-guided head regions, part-based heads, or graph/constraint formulations—so that status depends on the relative geometry rather than the incidental overlap of person and helmet boxes [36,37,38]. Segmentation-based or hybrid detect-then-segment designs offer finer boundary cues but can increase the latency and annotation cost [39]. Robustness across projects and cameras remains challenging due to domain shift in garments, backgrounds, and viewpoints; to improve generalization, works have explored extensive augmentation (color/blur/weather/copy-paste), synthetic PPE generation, and adaptation/regularization (self-training, style/feature alignment) [40,41,42]. Even so, evaluations often underrepresent minute-long, occlusion-rich videos typical of active sites, where severe truncation and repeated re-appearances accumulate detection errors [43].

### 2.2. Object Tracking Algorithms

Compared with generic pedestrian tracking, monitoring helmet wearing on active construction sites poses domain-specific difficulties: workers undergo long, heavy occlusions, operate in crowded scenes with visually similar PPE, and appear in minute-long videos with re-entries and lighting changes. Most systems adopt a tracking-by-detection paradigm in which per-frame detections are linked across time [44]. DeepSORT-style trackers couple a linear-Gaussian motion model with Hungarian one-to-one assignment and a global appearance embedding [45]. These design choices explain both strengths and failure modes: the constant-velocity prior stabilizes short gaps but is violated by abrupt human maneuvers, while the Hungarian solver commits globally based on a noisy cost matrix, so when detections are partially overlapped or missing, near-ties in IoU/appearance can swap identities at crossings. ByteTrack-like strategies that anchor association on high-confidence detections reduce sensitivity to low-quality boxes, but thresholding suppresses genuine but low-score observations during blur/occlusion, creating track fragmentation that later promotes switches on re-appearance [46]. In addition, re-ID embeddings trained on everyday attire exhibit a domain gap in PPE-rich scenes: the head ROI is small, salient colors are shared across helmets/vests, and background clutter reduces inter-class separability, which lowers appearance discrimination after short misses [47]. Taken together, identity errors emerge not because these trackers are “insufficient”, but because their probabilistic and assignment assumptions (linear motion, stationary appearance, reliable detections) are routinely violated on construction footage.

To mitigate these violations, recent work advances four complementary directions. (i) Stronger association metrics. By fusing geometric overlap with appearance and adding occlusion-aware gating, the cost surface becomes better conditioned, reducing tie cases that trigger Hungarian misassignments under partial visibility [48]. (ii) Sequence-level modeling. Tracklet linking, short-window temporal smoothing, or graph/RNN aggregation explicitly propagate evidence across frames, repairing gaps created when detectors down-score blurred/occluded heads [49]. (iii) PPE-aware re-identification. Head/upper body-focused embeddings or part cues increase the between-identity margin in scenes where full-body clothing is homogeneous or safety gear is similar, improving post-occlusion re-association [50]. (iv) Real-time efficiency. Lightweight feature extractors and streamlined association preserve the above gains within edge latency budgets common to site deployments [51]. Parallel to these, fuzzy-logic controllers treat IoU and appearance similarity as uncertain linguistic variables and adaptively re-weight them when either cue is unreliable, thereby smoothing decisions near ambiguous boundaries [52]; auxiliary cues such as head/pose keypoints can further constrain association by injecting geometric consistency when boxes are truncated [53]. Despite progress, many evaluations still emphasize short clips with mild occlusion, so accumulated errors from long disappearances and re-entries remain under-measured; in practice, identity continuity is the dominant failure mode precisely because assumption violations persist over long horizons.

## 3. Methodology

**Figure 2:**
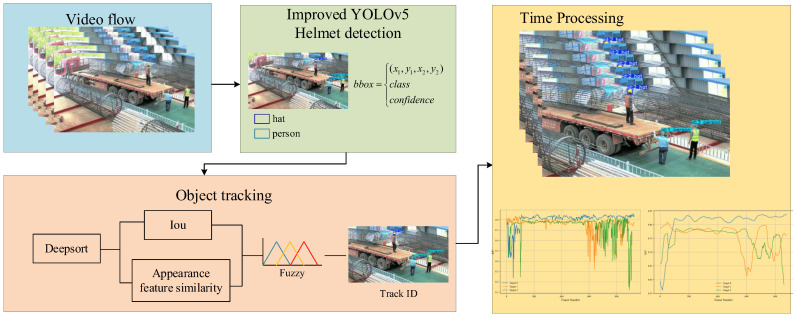
Overall framework of the algorithm process.

This section details the core technical methods for helmet detection and worker tracking on construction sites, with a specific focus on the design and functionality of each module. Figure 2 shows the overall framework of the algorithm proposed in this paper, which mainly consists of two key parts: (i) helmet detection and worker tracking based on the improved YOLOv5, and (ii) the helmet–worker matching process.

First, the improved YOLOv5 model is used for helmet detection, with a particular emphasis on the SACA mechanism. This attention mechanism helps guide the model to focus more on the head region, enabling the accurate identification of helmet usage, which significantly improves the robustness and accuracy of helmet detection. Next, the DeepSORT object tracking algorithm, combined with fuzzy logic, was used for worker matching and continuous tracking. Especially in situations with highly similar targets or partial occlusion, this method demonstrates high flexibility and stability. Finally, to further enhance the model’s adaptability in dynamic environments, a temporal processing mechanism for multi-frame information integration was introduced. This smooths the tracking results, ensuring high accuracy and consistency throughout the detection and tracking process.

### 3.1. Helmet Detection Algorithm Design

#### 3.1.1. YOLOv5 Algorithm Overview

Since its release, the YOLOv5 algorithm has quickly become the industry standard in the field of object detection due to its exceptional speed, accuracy, and ease of use. The algorithm is fully developed in PyTorch and continues to be optimized with support from the community and ecosystem. As an end-to-end object detection method, YOLOv5 directly predicts the location and class information of bounding boxes through regression, enabling fast and efficient object detection [54].

**Figure 3:**
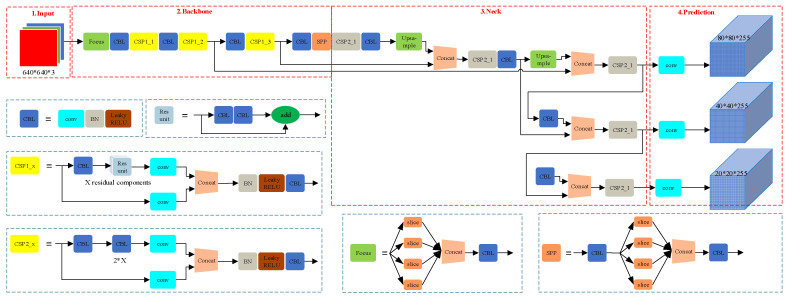
YOLOv5 algorithm network architecture diagram.

YOLOv5 offers significant advantages over traditional detection algorithms such as Faster R-CNN. Its model structure includes CSPDarknet as the backbone network and employs PANet to improve feature fusion capabilities, thereby enhancing the detection accuracy for small objects. Additionally, YOLOv5 improves the bounding box localization accuracy through an optimized prediction head, achieving excellent performance on several standard datasets (such as COCO and VOC) [55]. Extensive studies have investigated the YOLOv5 algorithm, and its structure is illustrated in Figure 3.

The network structure of YOLOv5 consists of three main parts: the backbone, the neck, and the head. The backbone network uses CSPDarknet, which improves the network’s features and computational efficiency through cross-stage partial connections. The neck structure employs PANet to fuse multiscale features, enhancing the detection accuracy. The head is responsible for the final bounding box regression and class prediction. The backbone network of YOLOv5 extracts deep features from images through multi-level convolution and pooling operations. The PANet structure in the neck fuses features from different scales through path aggregation to capture details of objects of various sizes. The head structure generates the final detection results including the class and position of the objects [56,57].

Despite the outstanding performance of YOLOv5 in many scenarios, traditional YOLO models still face challenges in certain complex environments, especially in cases of target occlusion and low-resolution images. Therefore, incorporating emerging attention mechanisms to improve YOLOv5’s performance, particularly in the detection of small objects such as helmets, is of significant practical importance. To address this, this paper introduces the SACA based on YOLOv5. The goal is to further enhance the model’s performance in complex environments through the improved attention mechanism, particularly in improving detection accuracy under occlusion and low contrast scenarios.

#### 3.1.2. SACA

**Figure 4:**
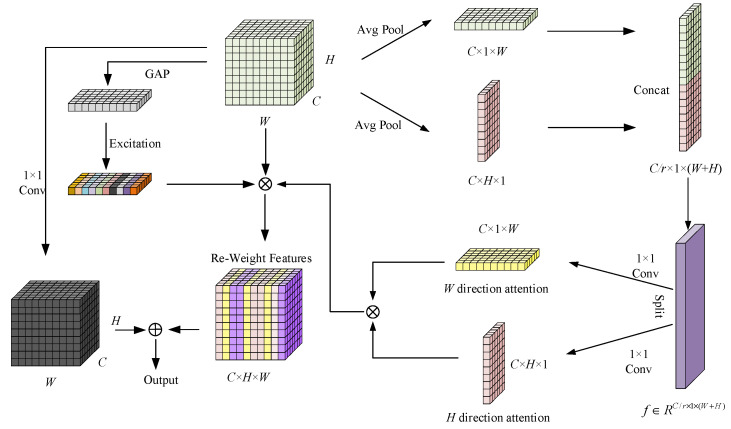
SACA structure diagram.

SACA enhances the accuracy of object detection by adaptively adjusting the attention regions in both spatial and channel dimensions, particularly improving the performance in occlusion and complex background scenarios. Unlike traditional attention mechanisms, SACA dynamically calculates the weights for both spatial and channel dimensions and incorporates non-local feature information to better focus the model on key regions. The algorithm flow is shown in Figure 4.

First, SACA performs adaptive pooling operations on the feature map in both the horizontal and vertical directions, generating feature maps at different scales. Given an input feature map $X\in R^{C\times H\times W}$, horizontal pooling produces C×H×1 features and vertical pooling produces C×1×W features. After lightweight convolutional operations, both pooled features are broadcast back to the original size C×H×W to generate spatial attention weights, ensuring that long-range dependencies in both horizontal and vertical directions are effectively modeled. The pooled feature maps are then fused through convolution operations to generate a spatial attention map. This process is represented as follows:

(1) $$x_{h}=\mathrm{AdaptiveAvgPool}2d\left(x\right)$$

(2) $$x_{w}=\mathrm{AdaptiveAvgPool}2d(x).permute(0,1,3,2)$$

(3) $$y=Conv1([x_{h},x_{w}])$$

Next, the spatial attention maps $a_{h}$ and $a_{w}$ are generated through convolution operations as follows:

(4) $$a_{h}=sigmoid\left({Conv}_{h}\left(x_{h}\right)\right)$$

(5) $$a_{w}=sigmoid({Conv}_{w}(x_{w}))$$

Then, SACA calculates the channel attention by applying global average pooling and convolution operations to obtain the channel weights as follows:

(6) $$a_{c}=sigmoid\left({Conv}_{c}\left(\mathrm{GlobalAvgPool}\left(x\right)\right)\right)$$

To further enhance the perception of global context, SACA introduces a non-local attention mechanism. Traditional local attention typically focuses only on local features, and in scenarios with target occlusion or complex backgrounds, local features often fail to provide sufficient contextual information. To address this issue, SACA integrates global information, enabling the model to capture long-range dependencies, thereby effectively avoiding false detections caused by insufficient local information. Finally, SACA generates the final output feature map by fusing spatial, channel attention, and non-local features as follows:

(7) $$O=X\cdot a_{h}\cdot a_{w}\cdot a_{c}+N$$

where X is the input feature map, $a_{h}$, $a_{w}$, and $a_{c}$ are the spatial, vertical, and channel attention maps, respectively, and N is the global context feature obtained through non-local attention.

### 3.2. Multi-Object Tracking Algorithm Design

**Figure 5:**
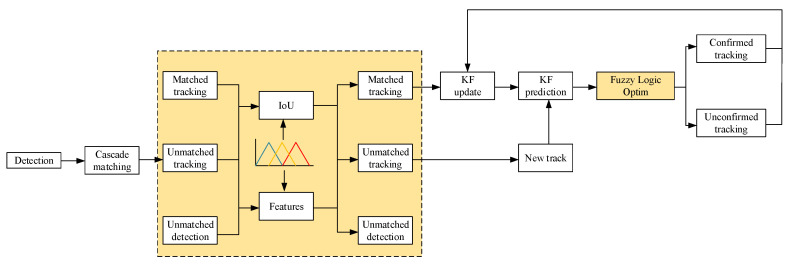
DeepSORT object tracking algorithm with fuzzy logic integration.

This paper proposes an improved particle tracking algorithm based on DeepSORT to more accurately track targets in challenging real-world scenarios such as occlusion and rapid motion. The improved DeepSORT structure is shown in Figure 5, with the yellow section representing the improvements. In the original DeepSORT, the algorithm uses a deterministic matching method (IoU matching) and a simple appearance feature extractor. While the algorithm performs well in many scenarios, it struggles in cases of occlusion, fast motion, target re-identification, and unreliable detection. These limitations lead to frequent tracking errors in high-dynamic environments such as incorrect labeling and target loss.

To address these challenges, we introduced a fuzzy logic-based optimization method into the DeepSORT framework. Specifically, we integrated a fuzzy inference system into the target matching process to improve the robustness against occlusion and motion variations. The fuzzy logic component not only uses traditional geometric features (IoU), but also incorporates appearance similarity to evaluate the matching degree between targets. It adjusts the matching process based on uncertain or ambiguous situations. By introducing fuzzy rules, the system can more flexibly adapt to uncertain conditions, maintaining the identity of targets even when their appearance changes or they are partially occluded.

The fuzzy logic-based improved structure is as follows:

(a) Fuzzy IoU matching

In the original DeepSORT, the IoU matching threshold is fixed. This method can lead to incorrect matches, particularly when the overlap between targets is small, but their appearance features are very similar. To improve this, we introduced fuzzy logic to dynamically adjust the IoU matching threshold.

We used fuzzy inference to adjust the IoU matching threshold. Through the following formula, we combined the IoU value and appearance similarity to determine the flexibility of the match:

(8) $$\mu_{match}(IoU,S)=\mu_{IoU}\left(IoU\right)\cdot \mu_{\mathrm{appearance}}(S)$$

where $\mu_{IoU}(IoU)$ is the fuzzy membership degree calculated based on the IoU value, and $\mu_{\mathrm{appearance}}(S)$ is the fuzzy membership degree calculated based on the appearance similarity of the target. IoU is the intersection over union ratio between the bounding boxes, and S is the similarity computed based on appearance features.

This formula dynamically adjusts the matching degree between targets using fuzzy logic, so that when the IoU value is low, if the appearance features are very similar, the targets can still be considered the same. Conversely, if the appearance features differ significantly, despite a high IoU, the targets may be considered different.

The fuzzy membership function for IoU is as follows:

(9) $$\mu_{loU}(IoU)=\left\{\begin{array}{cc} 1 & IoU>0.8\\ 0.7 & 0.5\leq IoU\leq 0.8\\ 0.3 & loU<0.5 \end{array}\right.$$

The fuzzy membership function for appearance similarity is as follows:

(10) $$\mu_{\mathrm{appearance}}(S)=\left\{\begin{array}{cc} 1 & S>0.9\\ 0.7 & 0.6\leq S\leq 0.9\\ 0.4 & S<0.6 \end{array}\right.$$

The specific thresholds in Equations (9) and (10) (e.g., IoU values of 0.5 and 0.8, and similarity values of 0.6 and 0.9) were selected based on the distribution analysis of the validation dataset, where they represent transition points between reliable and ambiguous matches. These choices are also consistent with prior studies [52], and can be flexibly adjusted for different application scenarios to balance between stability and sensitivity.

(b) Appearance similarity based on fuzzy inference

Traditional DeepSORT uses the appearance features of targets for matching, but relying solely on the direct comparison of appearance features may not be sufficient to cope with variations in real-world environments (e.g., lighting changes, occlusion, etc.). To address this, we introduced a fuzzy inference mechanism that evaluates appearance similarity through fuzzy rules.

By applying fuzzy logic, the tracking algorithm can better adapt to the complexities of the real-world, such as lighting changes and occlusion, thereby improving the performance and stability in dynamic environments.

We used cosine similarity to calculate the similarity between the appearance features of two targets:

(11) $$S={(f}_{1}\cdot f_{2})/(∥f_{1}∥∥f_{2}∥)$$

where $f_{1}$ and $f_{2}$ are the appearance feature vectors of target 1 and target 2, and ∥f∥ is the L2 norm of the vector.

By introducing fuzzy logic, the improved DeepSORT algorithm becomes more robust during the target matching process, enabling it to better handle complex situations in real-world environments such as lighting changes, occlusion, and target overlap. The fuzzy IoU matching and appearance similarity based on fuzzy inference make the object tracking more flexible and accurate, thereby enhancing the tracking performance and stability in dynamic environments.

### 3.3. Time Processing Method

To ensure the continuity and stability of helmet wearing status, we introduced a time-based processing method that integrates historical data from the previous frame. This method effectively corrects the issues of target loss and false detection caused by brief occlusion or misdetections, significantly improving the accuracy and stability of helmet wearing status. We recorded the helmet wearing status (HW status) for each frame and marked any lost frames as “NAN”. For false detections or targets temporarily lost, a sliding window was used to process the time-series data. If the number of frames in which the target appears is fewer than the set threshold, it is considered a false detection, and the target’s track is discarded.

The HW status is determined based on the comprehensive matching degree of IoU fuzzy membership and appearance similarity:

(12) $$HW\;state=\left\{\begin{array}{cc} 0 & \mathrm{IoU}>\mathrm{Threshold}\\ 1 & \mathrm{IoU}\leq \mathrm{Threshold} \end{array}\right.$$

By combining data from the set frames with information from the current frame, noise can be effectively filtered, and detection accuracy is improved, significantly enhancing the model’s stability in complex dynamic environments.

The size of the sliding window is a critical factor: smaller windows enable faster responsiveness but are more sensitive to noise, while larger windows provide more stable results by reducing false detections at the cost of increased latency. Therefore, the window size should be chosen according to the video frame rate and the complexity of the construction site environment, with a moderate window size offering a good balance between responsiveness and stability.

## 4. Dataset Description and Experimental Setup

### 4.1. Helmet Detection Dataset

This paper was primarily divided into two major parts: helmet detection and human tracking. Since the proposed algorithm is a general helmet detection method applicable to various scenarios, it is not limited to construction environments. Therefore, there is no need to specifically build a large dataset for construction site scenes. Additionally, the algorithm designed in this paper is also suitable for different application scenarios such as traffic and indoor work. As a result, we chose the publicly available SHWD dataset [58] for the experimental dataset.

**Figure 6:**
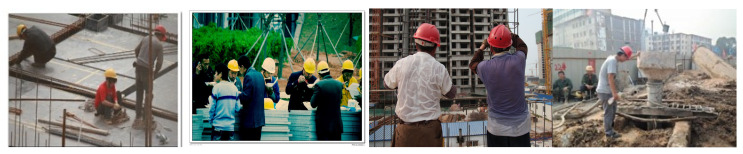
Examples from the SHWD dataset.

The SHWD dataset contains 7581 images, divided into two categories: images of workers wearing helmets and those without helmets, focusing on detecting the head region rather than the full body. The images in the dataset were sourced from Google and Baidu, covering a variety of scenarios including traffic, indoor work, and different types of construction environments. These images also include various lighting conditions (e.g., strong dim lighting) and helmets of different colors and shapes, fully demonstrating the method’s broad applicability. Figure 6 shows some example images from the dataset.

In addition to SHWD, three real construction site surveillance videos were further employed for validation, which contained challenges such as worker occlusion, posture changes, and new worker entries, thereby ensuring that the proposed method was also evaluated under complex real-world conditions. The videos is shown in Section 4.2.

### 4.2. Helmet-Wearing Worker Tracking

**Figure 7:**
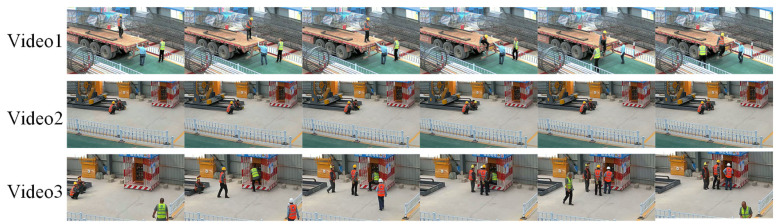
Example frames from Video1, Video2, and Video3.

To evaluate the accuracy of our algorithm, we selected three sets of real worker site monitoring video datasets, named Video1, Video2, and Video3. Figure 7 shows the cropped images from these three videos over time. The following is a detailed description of the content of these three videos. All videos were filmed using the same Hikvision camera, with a frame rate of 20 and a resolution of 1920 × 1080.

Video1: This video shows a worker wearing a helmet standing on a truck, while two other workers are working on the ground. The worker on the truck performs an exit action, showing a significant posture change, from sitting down to jumping off. Meanwhile, the two workers on the ground are in walking and standing positions, with one worker experiencing a brief occlusion during their walking process.

Video2: This video shows two workers sitting on the ground. One worker is wearing a helmet, while the other is not, with the helmet placed on the ground. The video clearly records the tracking changes of the same worker before and after wearing the helmet by showing the actions of putting on the helmet.

Video3: This video shows four workers working in front of an electrical distribution box. Initially, two workers wearing helmets are sitting, and they later join in the work at the distribution box. Another worker without a helmet starts working immediately, and the last worker, a new member, joins the operation. This video includes worker occlusion and a new worker joining the scene, increasing the complexity of the tracking task.

### 4.3. Experimental Setup

The experimental environment for training on the SHWD dataset and target tracking is shown in Table 1.

After experimental tuning, the training settings were as follows: the number of epochs was set to 50, the batch size was 16, the initial learning rate was set to 0.001, and the IoU threshold was 0.5. The model parameters were optimized using the Adam optimizer.

## 5. Experimental Results

### 5.1. Helmet Detection Experimental Results

In this study, precision, recall, and mAP@0.5 were used as the evaluation metrics for helmet detection and multi-object tracking tasks, as described below:

First, precision was used to measure the proportion of true positive samples among all the samples classified as positive by the model. The formula for calculating precision is:

(13) Precision=TP/(TP+FP)

where TP represents the number of true positives and FP represents the number of false positives. A higher precision indicates a lower probability of misclassification when detecting positive class targets.

Next, recall was used to measure the proportion of correctly identified positive samples among all of the actual positive samples. The formula for calculating recall is:

(14) Recall=TP/(TP+FN)

where FN represents the number of false negatives. A higher recall indicates that the model is able to detect more positive class targets, but it may also result in more false positives.

To comprehensively evaluate the model’s detection performance, we used mAP@0.5 as the evaluation metric. mAP@0.5 is calculated based on the area under the precision–recall curve, where the IoU threshold was set to 0.5. The formula for calculating mAP@0.5 is:

(15) $$mAP@0.5=\frac{1}{N}\sum_{i=1}^{N}AP_{i}$$

where N represents the number of categories, and AP_i is the average precision for the i_th class. The average precision was obtained by calculating the area under the precision–recall curve. A higher mAP indicates that the model performs better across different scenarios, especially in terms of robustness and accuracy in complex environments.

**Figure 8:**
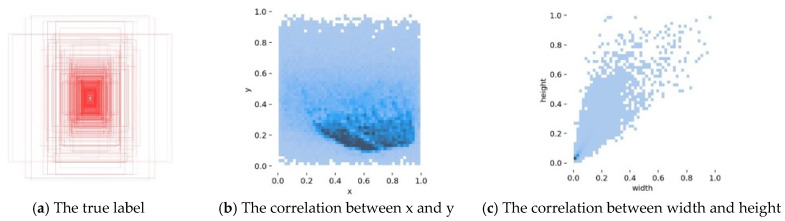
Internal structure of the SHWD dataset.

In this paper, the results obtained from training the proposed improved YOLOv5 algorithm on the SHWD dataset are shown in Figure 7. Figure 8a displays all the ground truth bounding boxes, while Figure 8b,c show that the height and width of the ground truth boxes were smaller than the height and width of the image, demonstrating the validity of the training.

**Figure 9:**
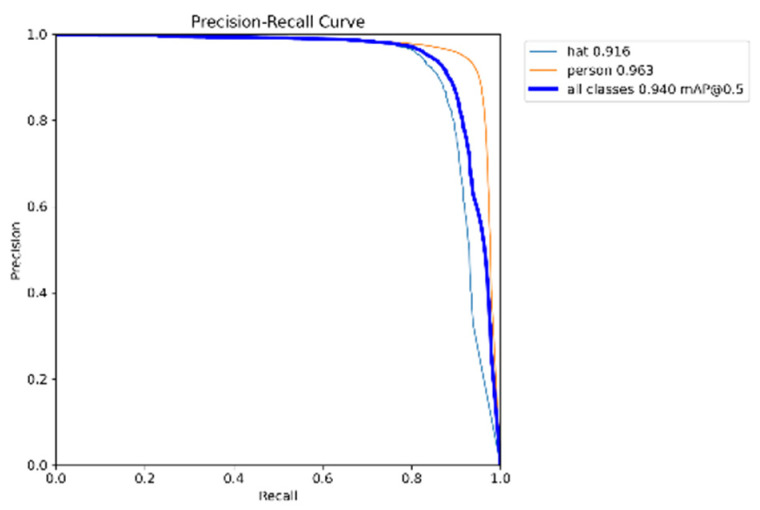
PR curve of model training.

Figure 9 shows the PR curve obtained from the improved YOLOv5 model we designed. As can be seen, the model achieved 91.6% accuracy for helmet detection, 96.3% accuracy for non-helmet detection, and an overall accuracy of 94.0%. This indicates that the model is particularly well-suited for detecting unsafe behaviors related to the absence of helmets.

To comprehensively evaluate the performance of the proposed model, it was compared not only with several recent YOLO algorithms, but also with typical attention-based variants of YOLOv5 such as SE, CBAM, and ECA. All algorithms were trained for 50 epochs, with a batch size of 16, to ensure fairness in the training conditions. The results are shown in Table 2. It can be observed that the proposed method performed excellently on the mAP@0.5 metric, achieving a value of 0.940, placing it in a leading position. Although the precision metric was not the highest, the model’s computational load had nearly not increased, and the number of parameters had not significantly grown based on the improvements made to YOLOv5. Maintaining such excellent performance represents a significant advancement. In addition, compared with the SE, CBAM, and ECA attention modules, the proposed SACA achieved a higher mAP and recall, showing its stronger capability in handling small targets and occlusion in construction site scenarios. This result fully validates the effectiveness of the introduced SACA, demonstrating that it can improve the detection accuracy while effectively controlling the computational resource consumption.

**Figure 10:**
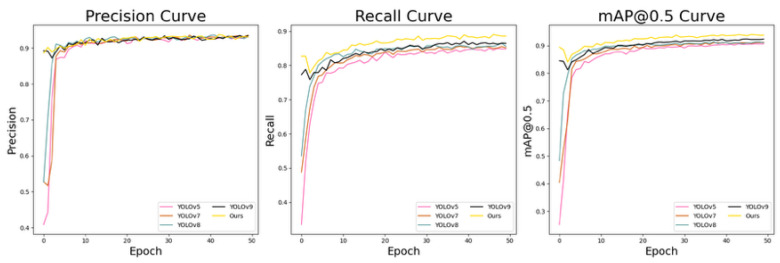
Comparison of three metrics for various algorithms, from left to right: precision, recall, and mAP@0.5.

Figure 10 presents a comparison of the three metrics for our improved YOLOv5 algorithm and several typical algorithms from Table 2. From the results, it can be seen that while our algorithm did not show a significant advantage in precision, it consistently led in both recall and mAP@0.5. This indicates that our model was more effective at correctly identifying instances.

In addition to accuracy, efficiency metrics were also evaluated to verify the real-time applicability of the proposed method. Table 3 summarizes the comparison of training time, inference speed (FPS), and GPU utilization with the baseline YOLOv5s and lightweight variants. The results showed that the proposed YOLOv5s + SACA achieved 28 FPS on an NVIDIA RTX 4070 GPU, which is sufficient for real-time helmet detection in construction sites, while maintaining higher accuracy than the lightweight alternatives.

### 5.2. Multi-Object Tracking for Helmet Detection

**Figure 11:**
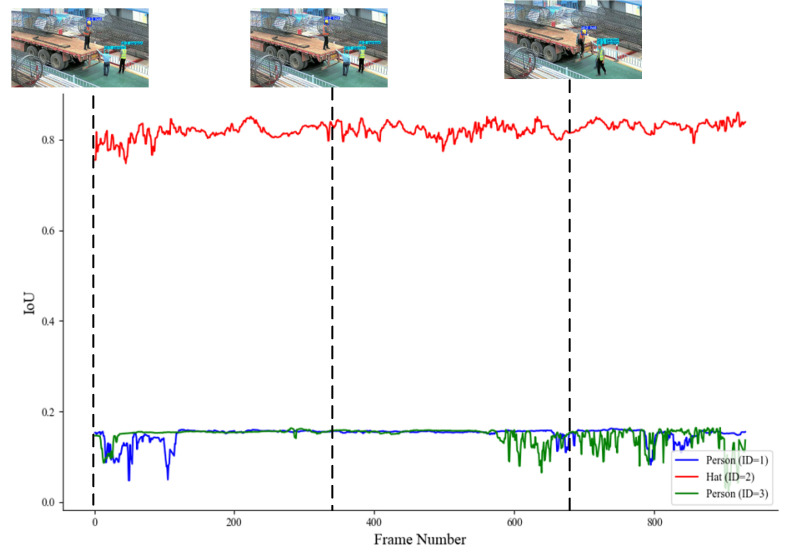
IoU matching in Video1.

Figure 11 shows the tracking results for the targets in Video1 using the proposed DeepSORT tracking algorithm with fuzzy logic integration. As previously mentioned, Video1 primarily demonstrates the detection of worker motion changes and occlusion phenomena. From Figure 10, it can be seen that for helmet detection, the IoU matching score remained consistently high, indicating that the method effectively tracked helmets in Video1.

Although in person detection the IoU matching score was lower during some periods due to occlusion of the worker, the overall tracking still fluctuated, and no misidentifications occurred. This suggests that the model can stably track whether workers are wearing helmets and demonstrates excellent robustness in complex scenarios.

**Figure 12:**
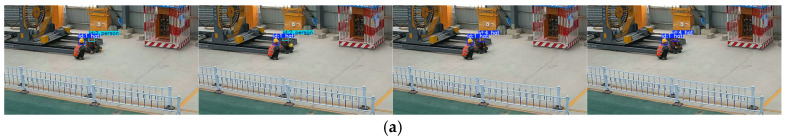

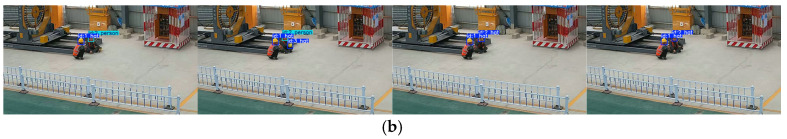
(**a**) Traditional DeepSORT tracking of helmet-wearing status in Video2. (**b**) Optimized DeepSORT tracking of helmet-wearing status in Video2.

In Video2, the process of a worker transitioning from not wearing a helmet to wearing one is demonstrated. Figure 12 compares the tracking performance between the traditional DeepSORT and the optimized DeepSORT on sample frames. It can be observed that the traditional DeepSORT encounters issues of missed helmet detection and ID switching errors when the worker puts on the helmet.

In contrast, the optimized DeepSORT, which incorporates fuzzy logic and appearance similarity, not only avoided the issue of missed helmet detection, but also accurately identified the changes in the helmet-wearing status of the same worker by introducing a temporal processing algorithm. This result fully demonstrates the superior performance and robustness of the optimized algorithm in complex scenarios.

To ensure temporal stability of helmet-wearing status across frames, a sliding-window mechanism was adopted to process the time-series data. The window size directly affects the trade-off between temporal smoothness and responsiveness. A smaller window can quickly reflect status transitions but is more susceptible to transient detection noise, while a larger window provides smoother predictions but introduces latency.

**Figure 13:**
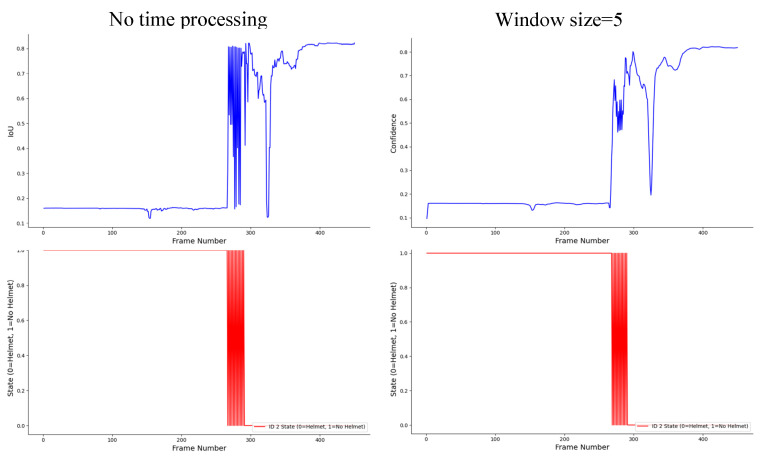

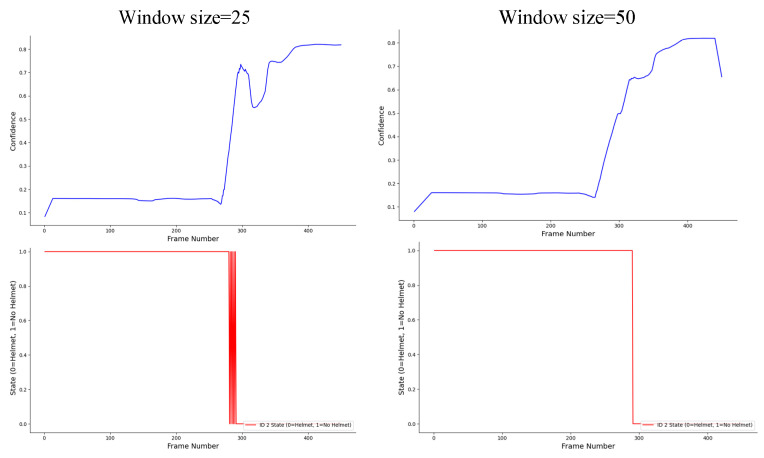
Comparison of temporal processing for helmet-wearing status detection of the second worker in Video2.

Through empirical experiments on 20 fps construction-site videos, we evaluated window sizes from 5 to 50 frames. As shown in Figure 13, a 20–30 frame window yielded the best balance, effectively filtering short-term noise while maintaining prompt responses to real changes. Hence, the optimal window length can be adapted according to the frame rate and the temporal dynamics of the specific application scenario.

From Figure 11, it is evident that without temporal processing, incorrect judgments occurred, especially during the process of wearing the helmet, where misidentifications repeatedly took place. However, after smoothing with a window function, this issue was significantly alleviated. When the window size was set to 50, false detections were almost entirely eliminated. Nevertheless, the window size must be carefully chosen; a window that is too large may lead to data loss, which could affect the accuracy of the results.

**Figure 14:**
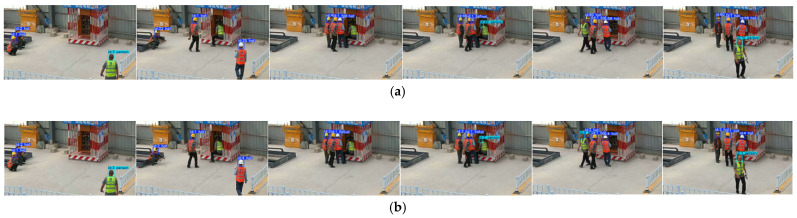
(**a**) Traditional DeepSORT tracking of helmet-wearing status in Video3. (**b**) Optimized DeepSORT tracking of helmet-wearing status in Video3.

For Video3, which was relatively more complex and includes long periods of worker occlusion as well as the introduction of other workers, the video effectively validates the tracking performance of the proposed algorithm in long-duration videos. Figure 14a,b show examples of multi-worker tracking using the traditional DeepSORT algorithm and the optimized DeepSORT algorithm, respectively. From Figure 14, it can be seen that the traditional DeepSORT algorithm encountered ID mismatches when new workers appeared and failed to recover the original worker’s ID during long occlusion periods. In contrast, the optimized DeepSORT algorithm, although still experiencing brief ID loss, was able to accurately recover the original worker’s ID after a short time, fully demonstrating the stability and robustness of the proposed algorithm in long-duration videos.

For multi-object tracking, we used the following three evaluation metrics to measure the overall performance of the tracking algorithm.

(a) MOTA (multiple object tracking accuracy)

MOTA takes into account false positives (FP), false negatives (FN), and identity switches (IDSw), and is calculated using the following formula:

(16) MOTA=1−(FN+FP+IDSw)/GT

where GT represents the total number of ground truth objects. A higher MOTA value indicates a lower probability of errors during tracking, signifying more accurate and stable tracking results.

(b) IDF1 (Identity F1 Score)

IDF1 is a metric that measures the performance of target identity recognition, combining the number of correctly identified target identities with the number of mismatches. The formula for calculating IDF1 is

(17) $$IDF1=\frac{2\times IDTP}{2\times IDTP+IDFP+IDFN}$$

where IDTP represents the number of correctly matched target identities, IDFP represents the number of incorrectly matched target identities, and IDFN represents the number of unmatched target identities.

(c) IDS (Identity Switches)

IDS refers to the number of times the model incorrectly switches the identity of a target during multi-object tracking. Identity switches cause instability in tracking, so a lower IDS value indicates better performance in maintaining target identity consistency. This metric does not have a fixed formula and is evaluated by calculating the number of target identity switches.

The comparison of the proposed algorithm with several advanced methods is shown in Table 4 and Table 5. To ensure fairness and better isolate the contributions of detection backbones and tracking strategies, we report the results under two settings: (i) fixing the detector (YOLOv5-SACA) while varying the tracker, and (ii) fixing the tracker (DeepSORT with fuzzy logic and temporal processing) while varying the detector. In addition, we included several representative models such as anchor-free detectors (CenterNet), transformer-based detectors (Deformable DETR), and joint detection–tracking methods (FairMOT).

From Table 4, where YOLOv5-SACA was fixed as the detector, different tracking algorithms achieved varying levels of performance. ByteTrack achieved the highest MOTA and IDF1 values (91.0% and 85.1%), but at the cost of increased computational complexity and GPU memory consumption, which may limit its deployment in resource-constrained construction environments. In contrast, the proposed DeepSORT with fuzzy logic and temporal processing achieved a slightly lower accuracy, but with the lowest number of identity switches (5), indicating more stable long-term tracking and a better trade-off between accuracy and efficiency.

From Table 5, where DeepSORT with fuzzy logic was fixed as the tracker, YOLOv9 and Deformable DETR achieved strong detection performance, slightly surpassing the proposed YOLOv5-SACA in MOTA and IDF1. However, YOLOv9 requires significantly more parameters and FLOPs, while transformer-based models like Deformable DETR introduce high inference latency, which restricts their real-time applicability. In contrast, the proposed YOLOv5-SACA achieved comparable accuracy with lower computational overhead and the lowest IDS value, making it more suitable for real-time helmet detection in complex construction site scenarios.

## 6. Conclusions and Discussion

### 6.1. Conclusions

This study explored the application of the multi-object tracking method based on YOLOv5 and DeepSORT in construction site helmet detection. This research found that combining the YOLOv5 detection model with the DeepSORT tracking algorithm demonstrated high accuracy in monitoring helmet-wearing status. By introducing the SACA and temporal processing algorithm, the model’s stability and robustness in complex scenarios were enhanced. Experimental results showed that the proposed model outperformed existing multi-object tracking algorithms in terms of the MOTA, IDF1, and IDS metrics, with significant advantages, especially in long-duration videos and occlusion scenarios. In particular, the introduction of the SACA further enhanced the detection accuracy, proving its effectiveness in handling dynamic worker state changes.

However, there are still some limitations in the proposed method. Although the introduction of the temporal processing algorithm reduced misdetections and identity switches when tracking multiple workers, there may still be occasional loss and misjudgment in extreme occlusion or complex backgrounds. Additionally, while the SACA mechanism significantly improved the detection accuracy, its computational overhead also increased. Future work could focus on further optimizing the computational efficiency, especially for applications on large-scale datasets. Future research may also concentrate on combining temporal processing with other deep learning methods (e.g., Transformer models) to further enhance detection and tracking performance in multi-worker scenarios.

### 6.2. Discussion

The experimental results demonstrate that the proposed method achieved excellent performance in construction site helmet detection tasks, particularly in long-duration videos and occlusion scenarios, showing a clear advantage over existing algorithms. The proposed approach integrates the detection capability of YOLOv5 with the DeepSORT tracking algorithm, fully leveraging the strengths of deep learning in both object detection and multi-object tracking. By incorporating the self-adaptive coordinate attention (SACA) mechanism, the model effectively captured subtle variations in workers’ helmet-wearing behavior, thereby improving the detection accuracy. Furthermore, the temporal processing algorithm enhanced the model stability when handling long video sequences, reducing misdetections and identity switches. In addition to the SHWD dataset, the proposed method was also validated on three real construction site surveillance videos, demonstrating its effectiveness and robustness in complex, real-world environments. As large-scale public construction site datasets remain limited, we supplemented SHWD with self-collected footage for training and validation. Future work will focus on constructing larger and more diverse datasets to enable more comprehensive evaluation and further generalization analysis.

However, despite the significant progress achieved in multi-object tracking, there are still some challenges. First, detection under extreme occlusion remains a difficult problem, especially when multiple targets overlap or move rapidly. In such cases, the detector may fail to generate reliable bounding boxes, causing both IoU and appearance similarity features to become unreliable. Meanwhile, the Kalman filter predictions may drift during long-term occlusion, and once the target reappears, it is sometimes assigned a new identity, leading to false detections and ID switches. Second, while the SACA mechanism effectively improves the detection accuracy, it also increases the computational complexity of the model. Future research could therefore explore more efficient attention mechanisms or optimization strategies to reduce the computational burden. Finally, this method primarily relies on static video frames for detection and tracking. Future research could investigate video-level detection frameworks or temporal modeling approaches (e.g., Transformer-based methods) to further enhance the model’s performance in real-world applications.

In future work, deep learning-based multi-object tracking methods can be further integrated with self-supervised and semi-supervised learning techniques to reduce dependence on large-scale labeled datasets. In construction site environments, where workers’ helmet-wearing behaviors are highly dynamic, employing self-supervised learning for model pre-training can effectively enhance the adaptability and generalization across diverse scenarios. Moreover, for cross-frame target tracking, future research could incorporate richer temporal information and reinforcement learning strategies to further improve the accuracy and stability of detection and tracking. With these advancements, helmet detection systems can be more effectively applied to real-world construction environments, contributing to more efficient and reliable safety management systems, ultimately strengthening worker protection.

## Figures and Tables

**Table 1:** Experimental platform and environment.

Component	Parameters
CPU	13th Gen Intel(R) Core(TM)i7-14700KF
RAM	32 G
GPU	NVIDA GeForce RTX 4070 12 GB
Programing language	Python 3.8
Deep learning framework	PyTorch 2.3.1
CUDA	11.8

**Table 2:** Comparison of indicators of helmet detection by different YOLO algorithms.

Model	Precision	Recall	mAP@0.5
YOLOv5	0.931	0.849	0.907
YOLOV7	**0.935**	0.852	0.912
YOLOV8	0.933	0.857	0.919
YOLOV9	0.934	0.864	0.924
YOLOv5 + SE	0.936	0.872	0.926
YOLOv5 + CBAM	0.938	0.878	0.931
YOLOv5 + ECA	0.937	0.874	0.928
Improved YOLOv5	0.924	**0.890**	**0.940**

**Table 3:** Comparison of the accuracy and efficiency metrics (training time, FPS, GPU usage) across models.

Model	Params (M)	FLOPs (G)	Inference FPS (RTX 4070)	mAP@0.5
YOLOv5s (baseline)	7.2	16.5	34	0.907
YOLOv5s + CBAM	7.8	17.9	30	0.931
YOLOv5s + SACA	8.0	18.2	28	0.940
YOLOv8n	3.2	8.7	40	0.919

**Table 4:** Tracking performance comparison with YOLOv5-SACA as the detector.

Tracker	MOTA	IDF1	IDS
StrongSORT	85.2%	80.3%	12
BoT-SORT	88.7%	83.6%	10
ByteTrack	91.0%	85.1%	9
OC-SORT	87.2%	82.0%	11
FairMOT (joint MOT)	86.1%	81.5%	14
DeepSORT	86.3%	82.4%	8
DeepSORT + Fuzzy + Time processing	90.5%	84.2%	5

**Table 5:** Tracking performance comparison with DeepSORT + fuzzy logic as the tracker.

Detector	MOTA	IDF1	IDS
YOLOv5 (baseline)	85.1%	81.0%	11
YOLOv8	88.2%	83.5%	9
**YOLOv9**	**91.3%**	**85.6%**	8
CenterNet (anchor-free)	87.0%	82.2%	12
Deformable DETR (transformer)	90.1%	84.5%	10
**YOLOv5-SACA (proposed)**	90.5%	84.2%	**5**

## Data Availability

No new data were created or analyzed in this study. Data sharing is not applicable to this article.
